# Laryngeal transplantation: from experimental feasibility to multidisciplinary clinical reality

**DOI:** 10.3389/fsurg.2026.1833564

**Published:** 2026-08-27

**Authors:** Lu Zeng, Longhao Wang

**Affiliations:** 1 Department of Anesthesia, Surgery and Intervention Center‌, West China Hospital, Sichuan University, Chengdu, Sichuan, China; 2 Department of Otorhinolaryngology Head and Neck Surgery, West China Hospital, Sichuan University, Chengdu, Sichuan, China

**Keywords:** bioengineering, chronic rejection, ethical considerations, functional recovery, immunosuppression, laryngeal transplantation, neural reinnervation, oncologic safety

## Abstract

Laryngeal transplantation represents one of the most complex forms of vascularized composite allotransplantation (VCA), aiming to restore respiration, phonation, and swallowing in patients with irreversible laryngeal dysfunction. Over the past decades, systematic preclinical research—including refinement of large-animal and rodent models, organ preservation optimization, and elucidation of immunological mechanisms—has laid the scientific foundation for clinical translation. Advances in static cold storage, emerging machine perfusion technologies, and biomarker-based precision monitoring have further improved graft viability and rejection surveillance. Clinically, since the first successful human laryngeal transplantation in 1998, approximately 16 cases worldwide have demonstrated the feasibility of long-term graft survival and meaningful functional recovery. More recently, indications have expanded from trauma-related defects to carefully selected oncologic patients under rigorous immunological and ethical oversight. Surgical progress has focused on microvascular reconstruction, selective neural reinnervation, and airway stabilization, while postoperative management emphasizes individualized immunosuppression, structured rehabilitation, infection prophylaxis, and lifelong surveillance for rejection and malignancy. Nonetheless, major challenges persist, including chronic rejection, variability in functional outcomes, oncologic safety under immunosuppression, donor scarcity, and unresolved ethical issues in a non–life-saving transplant context. Future directions center on integrating multi-omics biomarkers, machine-learning–based monitoring, bioengineered scaffolds, and personalized immunomodulation to enhance long-term graft function and safety. Overall, laryngeal transplantation has evolved from experimental feasibility to a clinically realistic reconstructive strategy, yet its broader application will depend on multidisciplinary innovation and rigorous ethical governance.

## 1 Introduction

Laryngeal transplantation is a highly complex form of vascularized composite allotransplantation (VCA) that aims to restore the entire laryngeal structure through microsurgical replacement of a damaged or absent larynx with a donor organ. The ultimate goal of this procedure is the simultaneous reconstruction of the three essential laryngeal functions—respiration, phonation, and swallowing. As a typical multi-tissue composite graft, laryngeal transplantation involves the integrated survival of mucosa, cartilage, muscle, and neural components, and critically depends on meticulous vascular reconstruction, precise neural anastomosis, and lifelong immunosuppressive therapy to ensure graft viability and functional integration (1, 2).Unlike solid organ transplantation, the success of laryngeal transplantation is not defined solely by graft survival, but rather by the coordinated restoration of complex physiological functions and meaningful improvement in recipient quality of life (3). These unique requirements render laryngeal transplantation one of the most technically demanding and academically challenging procedures within the VCA field.

In clinical practice, total laryngectomy remains the primary treatment for advanced laryngeal carcinoma and severe laryngeal destruction. However, conventional post-laryngectomy rehabilitative strategies—including permanent tracheostomy, artificial laryngeal prostheses, and esophageal speech—while effective in sustaining life, fail to simultaneously achieve natural respiration, high-quality phonation, and safe swallowing (4). Patients frequently experience poor voice quality, increased aspiration risk, and substantial psychosocial burden, leading to long-term impairment in quality of life. This unmet clinical need has persisted for decades without a definitive solution. In this context, laryngeal transplantation, by preserving the native anatomical integrity and physiological complexity of the larynx, is regarded as the only reconstructive approach with the potential to fully restore social function and overall quality of life in laryngectomized patients (5, 6). Consequently, it represents the most advanced and comprehensive strategy in functional laryngeal reconstruction.

The clinical realization of laryngeal transplantation was not achieved abruptly, but rather emerged from decades of systematic preclinical research. In the 1960s, orthotopic laryngeal transplantation models in dogs first demonstrated the technical feasibility of laryngeal vascular anastomosis. These pioneering studies not only established fundamental criteria for graft survival and functional assessment, but also identified two major barriers to successful transplantation: ischemia–reperfusion injury and acute immune rejection (7, 8). Subsequent investigations explored laryngeal replantation and reinnervation strategies, demonstrating that partial sensory and motor function recovery could be achieved under experimental conditions. Collectively, these studies highlighted that laryngeal transplantation is not merely a surgical endeavor, but rather a multidisciplinary enterprise integrating microsurgery, transplant immunology, and neurobiology, which directly stimulated advances in organ preservation techniques, immunosuppressive regimens, and perioperative management (9). By the 1990s, refinements in microsurgical nerve anastomosis—particularly involving the superior and recurrent laryngeal nerves—established the theoretical and experimental basis for restoring laryngeal sensory and motor functions in human recipients (10, 11).

Despite substantial progress in preclinical research, the clinical translation of laryngeal transplantation followed a prolonged and challenging trajectory. In 1969, a Belgian surgeon performed the first attempted human laryngeal allotransplantation in a 62-year-old patient with laryngeal carcinoma. Although the surgical procedure itself was technically successful, the patient died several months later due to tumor recurrence. This outcome had a profound impact on the field, leading to an almost two-decade stagnation in clinical exploration (12, 13). It was not until 1998 that Strome and colleagues in the United States achieved the first successful vascularized human laryngeal transplantation. The recipient regained phonation shortly after surgery, and the transplanted larynx maintained partial physiological function for more than 14 years, thereby providing definitive proof of long-term feasibility in humans (14). Nevertheless, this landmark achievement simultaneously brought ethical considerations to the forefront. As laryngeal transplantation involves a non–life-saving organ, the balance between lifelong immunosuppression-related risks and anticipated quality-of-life benefits remains a central ethical and clinical dilemma requiring careful evaluation (15, 16).

In recent years, laryngeal transplantation has entered a renewed phase of development, driven by the maturation of VCA concepts, advances in immunosuppressive management, and the establishment of multidisciplinary care models. In April 2023, the West China Hospital team successfully performed the first laryngeal transplantation in Asia, followed by three additional procedures within one year (17). Clinical follow-up demonstrated effective restoration of speech function and significant improvement in swallowing ability in all recipients. These achievements not only indicate improved reproducibility and perioperative management of laryngeal transplantation, but also reflect a substantial advancement in China's capabilities in high-complexity organ transplantation and composite tissue reconstruction.

**Figure 1:**
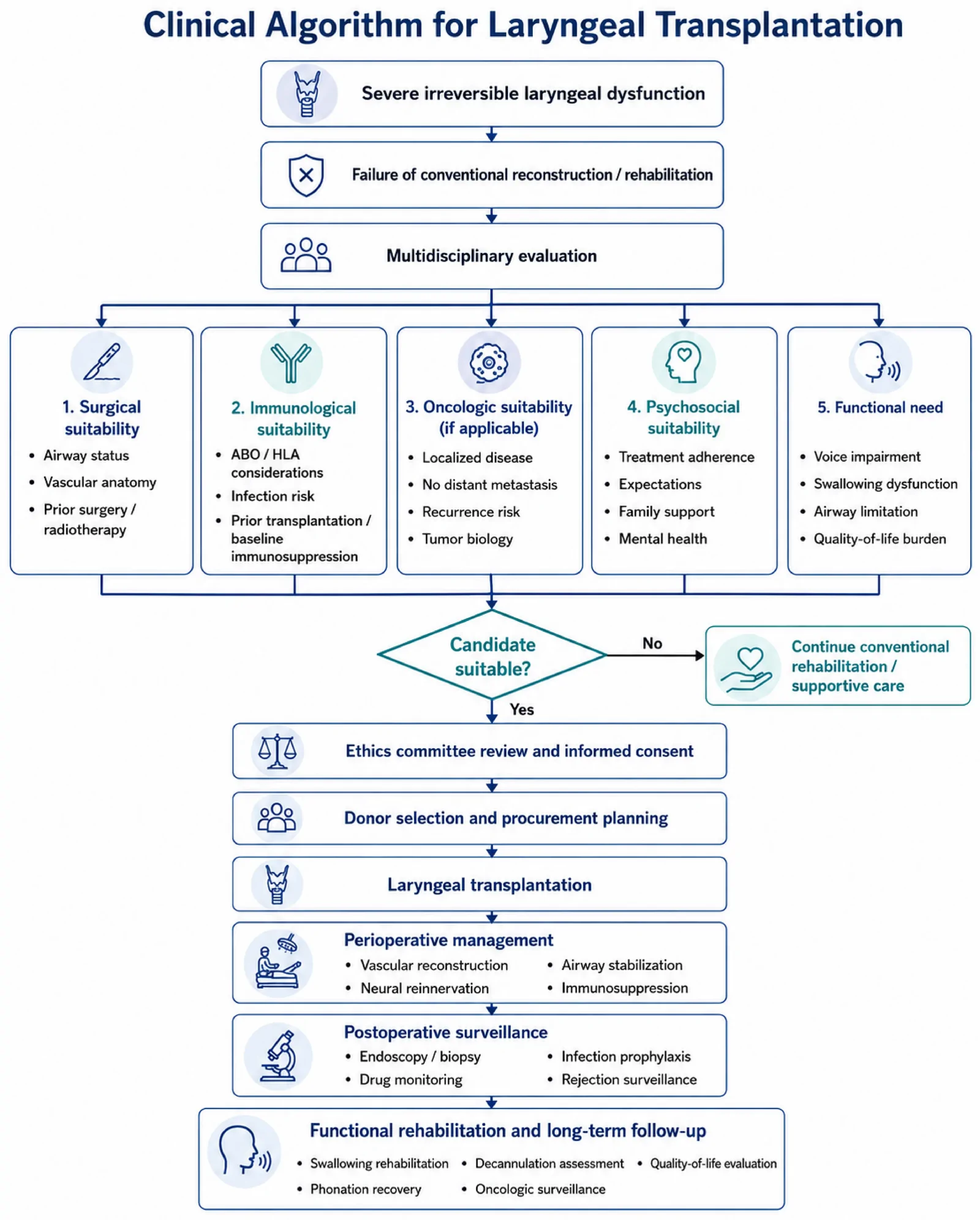
Proposed clinical algorithm for laryngeal transplantation. This algorithm illustrates a structured multidisciplinary pathway for candidate selection, perioperative management, and long-term follow-up in laryngeal transplantation. Patients with severe irreversible laryngeal dysfunction who fail conventional reconstruction or rehabilitation should undergo comprehensive multidisciplinary evaluation, including surgical suitability, immunological risk, oncologic status when applicable, psychosocial readiness, and functional need. Candidates considered unsuitable should continue conventional rehabilitation or supportive care. Suitable candidates should proceed to ethics committee review, informed consent, donor selection, procurement planning, and transplantation. Perioperative management requires coordinated vascular reconstruction, neural reinnervation, airway stabilization, and individualized immunosuppression. Postoperative care should include endoscopic and histological surveillance, therapeutic drug monitoring, infection prophylaxis, and rejection monitoring. Long-term management focuses on swallowing rehabilitation, phonation recovery, decannulation assessment, oncologic surveillance, and quality-of-life evaluation. ABO, ABO blood group system; HLA, human leukocyte antigen.

Although the existing literature demonstrates the technical feasibility of laryngeal transplantation, the overall quality of evidence remains limited. Most available data are derived from isolated case reports, small single-center experiences, and preclinical studies, with substantial heterogeneity in indications, donor selection, surgical techniques, immunosuppressive protocols, rehabilitation strategies, and outcome assessment. Therefore, current conclusions should be interpreted cautiously. In particular, long-term graft survival and functional recovery cannot be evaluated solely on the basis of successful individual cases, because negative outcomes, incomplete recovery, chronic rejection, and unsuccessful attempts may be underreported. In addition, several key clinical questions remain unresolved, including the optimal timing of transplantation, standardized criteria for candidate selection, objective assessment of voice and swallowing outcomes, and the acceptable risk threshold for lifelong immunosuppression in a non–life-saving procedure. Thus, the present review not only summarizes the development of laryngeal transplantation but also critically evaluates the limitations and translational uncertainties of the current evidence. Through this analysis, we seek to provide a rational framework to support the standardized development and responsible clinical translation of this emerging field. The proposed clinical algorithm for laryngeal transplantation was shown in Figure 1.

## 2 Methods

A narrative literature search was performed in PubMed/MEDLINE, Embase, Web of Science, Scopus, and Google Scholar from database inception to May 2026. Search terms included “laryngeal transplantation,” “larynx transplantation,” “laryngeal allotransplantation,” “vascularized composite allotransplantation,” “laryngeal reinnervation,” “organ preservation,” “immunosuppression,” “functional recovery,” and “oncologic reconstruction.” Reference lists of relevant articles were also manually screened. Eligible literature included preclinical studies, clinical case reports or series, reviews, and ethical or translational discussions related to laryngeal transplantation. Given the small number of cases and heterogeneity of study designs, interventions, and outcomes, the evidence was synthesized narratively rather than quantitatively.

## 3 Results and discussion

### 3.1 Preclinical advances in laryngeal transplantation

Laryngeal transplantation represents one of the most challenging frontiers in reconstructive surgery and offers a potential solution for patients with irreversible laryngeal dysfunction by enabling simultaneous restoration of anatomical integrity and physiological function. The feasibility of its clinical translation is highly dependent on rigorous and systematic preclinical research. Over the past several decades, investigations focusing on the development of animal models, optimization of organ preservation strategies, and refinement of immunosuppressive regimens have progressively addressed the key anatomical, immunological, and technical barriers inherent to laryngeal transplantation. Collectively, these efforts have laid a solid foundation for bridging the translational gap from experimental feasibility to clinical application.

#### 3.1.1 Animal models: the cornerstone of translational research

Preclinical research in laryngeal transplantation relies heavily on appropriate animal models to replicate the complex anatomy, physiological functions, and immunological characteristics of the human larynx. Different species offer distinct advantages depending on specific research objectives, and together they form a comprehensive experimental framework for advancing laryngeal transplantation. Among these, large-animal models play a particularly critical role in refining surgical techniques and investigating immunological mechanisms.

Porcine models, owing to their close resemblance to humans in laryngeal anatomy, vascular architecture, and immune responses, are widely regarded as optimal platforms for optimizing surgical strategies and perioperative management in laryngeal transplantation. Studies using Minnesota miniature pigs have demonstrated that under tacrolimus-based immunosuppressive protocols, reconstruction of graft perfusion via cervical vascular anastomosis combined with strict control of cold ischemia time (median approximately 3 h) resulted in graft survival rates as high as 87.5% at 14 days post-transplantation (18, 19). These findings not only confirmed the feasibility of vascularized laryngeal transplantation in large-animal models but also provided critical insights into donor–recipient vessel selection, anastomotic sequencing, and ischemia management relevant to clinical practice.

Beyond surgical feasibility, porcine models have been instrumental in elucidating the local immunological microenvironment of laryngeal allografts. Histological and immunohistochemical analyses revealed abundant expression of major histocompatibility complex (MHC) class II molecules within the laryngeal mucosa, accompanied by dense infiltration of dendritic cells and CD3⁺ T lymphocytes. The spatial distribution of these immune components closely mirrors the mucosal immune surveillance architecture observed in humans (9, 20). These findings suggest that the laryngeal mucosa constitutes a critical immunological target in allograft rejection and may play a pivotal role in chronic rejection and long-term functional deterioration, thereby providing a rationale for the development of targeted immunomodulatory strategies.

In contrast, rodent models are limited by small vessel diameters and thus cannot fully recapitulate the microsurgical complexity encountered in clinical laryngeal transplantation. Nevertheless, they remain invaluable for high-throughput mechanistic studies and molecular-level investigations. In rat models, shunted laryngeal grafts achieved vascular patency rates of approximately 64%, and further analyses demonstrated upregulation of embryonic myosin heavy chain expression in denervated grafts. These observations suggest that neural input may influence graft adaptation by modulating muscle fiber phenotype, immune responses, and functional integration (21, 22). Such findings have provided important insights into the interplay between neural regulation, immune modulation, and muscle remodeling in transplanted larynges.

Functional reconstruction has been extensively investigated using feline and porcine models to explore mechanisms of laryngeal nerve regeneration and recovery of motor function. Experimental studies have consistently demonstrated that selective nerve anastomosis is a decisive determinant of functional outcomes. Targeted reconstruction involving the phrenic nerve or recurrent laryngeal nerve enabled approximately 75% of experimental animals to regain varying degrees of vocal fold motion (23, 24). These results underscore the critical importance of nerve selection and anastomotic precision and further emphasize that successful laryngeal transplantation should not be evaluated solely by graft survival, but rather by the extent of effective reinnervation and coordinated functional recovery (Table 1).

**Table 1:** Animal models used in preclinical laryngeal transplantation research.

Animal model	Primary purpose	Key findings	Major advantages	Key limitations
Canine (dog)	Early feasibility and surgical exploration	Demonstrated technical feasibility of orthotopic laryngeal transplantation; Identified ischemia–reperfusion injury and immune rejection as major barriers	Large airway size; Suitable for early microsurgical exploration	Ethical constraints; Limited immunologic characterization
Porcine (miniature pig)	Translational surgical and immunological studies	High short-term graft survival under tacrolimus-based immunosuppression; Close resemblance of laryngeal anatomy and mucosal immune architecture to humans	Anatomical and immunological similarity to humans; Suitable for vascular and preservation studies	High cost; Complex perioperative care
Rodent (rat)	Mechanistic and molecular studies	Demonstrated impact of denervation on muscle remodeling; Identified parathyroid hormone as a potential early biomarker of rejection	Low cost; Suitable for mechanistic and biomarker research	Small vessel caliber; Limited translational surgical relevance
Feline (cat)	Functional and neural reinnervation studies	Selective nerve anastomosis enabled partial recovery of vocal fold motion in a majority of models	Favorable laryngeal neuromuscular anatomy for reinnervation studies	Limited availability; Ethical and logistical constraints

#### 3.1.2 Organ preservation

Organ preservation is a critical determinant of graft viability and functional recovery in laryngeal transplantation. Unlike solid organs, the larynx is a vascularized composite tissue composed of mucosa, cartilage, skeletal muscle, and neural elements, each exhibiting distinct metabolic demands and ischemic tolerance, which places laryngeal transplantation within the broader framework of vascularized composite allotransplantation (VCA) (25, 26). This structural and biological heterogeneity renders the larynx particularly vulnerable to preservation-related injury and necessitates tailored preservation strategies beyond those established for conventional solid organ transplantation (27).

Preclinical studies have consistently demonstrated that cold ischemia time (CIT) exerts a profound impact on early graft survival and subsequent functional outcomes in laryngeal transplantation. In large-animal models, prolonged cold ischemia has been associated with mucosal edema, endothelial dysfunction, and microvascular thrombosis, ultimately compromising reperfusion and accelerating inflammatory injury (28, 29). Importantly, intrinsic laryngeal muscles and mucosal epithelium appear substantially more susceptible to ischemic damage than cartilaginous structures, underscoring the need for strict control of preservation duration.

Static cold storage using conventional preservation solutions, such as University of Wisconsin (UW) and histidine–tryptophan–ketoglutarate (HTK), remains the most widely employed approach in experimental laryngeal transplantation. These solutions effectively reduce metabolic demand and limit cellular swelling; however, they offer incomplete protection against ischemia-induced endothelial activation and microcirculatory collapse, particularly within the laryngeal mucosa (30, 31). As a result, preservation-related injury continues to represent a major barrier to optimal graft function.

Emerging evidence from vascularized composite allotransplantation research suggests that hypothermic machine perfusion may provide superior preservation compared with static cold storage. By maintaining continuous low-flow perfusion under hypothermic conditions, this approach better preserves endothelial integrity, attenuates ischemic apoptosis, and facilitates metabolic waste clearance (32, 33). Although direct data specific to laryngeal transplantation remain limited, extrapolation from facial and limb allograft models supports the potential of perfusion-based strategies to extend preservation time and improve early graft function (34).

Collectively, these findings highlight that successful laryngeal transplantation requires preservation protocols specifically adapted to the unique biological characteristics of the larynx. Continued refinement of preservation strategies, including optimization of cold ischemia thresholds and exploration of perfusion-based technologies, will be essential for improving graft reliability and enabling broader clinical translation (35) (Table 2).

**Table 2:** Organ preservation strategies in preclinical laryngeal transplantation.

Preservation strategy	Experimental context	Key observations	Advantages	Limitations
Static cold storage (UW solution)	Large-animal and rodent laryngeal transplantation models	Reduced metabolic demand and preserved basic graft viability; Incomplete protection of mucosal endothelium and microcirculation	Widely available; Clinically familiar	Limited protection against ischemia–reperfusion injury; Mucosal edema and endothelial dysfunction
Static cold storage (HTK solution)	Experimental VCA and laryngeal models	Comparable metabolic suppression to UW; Variable protection of microvascular integrity	Low viscosity; Ease of flush and storage	Inadequate prevention of microcirculatory collapse in composite tissues
Cold ischemia time (CIT) control	Porcine laryngeal transplantation models	Prolonged CIT associated with mucosal edema, endothelial injury, and microvascular thrombosis; Muscle and mucosa more vulnerable than cartilage	Directly improves early graft viability	Logistically demanding; Limited preservation window
Hypothermic machine perfusion	VCA models (face, limb); Extrapolated to larynx	Improved endothelial integrity, reduced apoptosis, and enhanced metabolic waste clearance compared with static storage	Potential extension of preservation time; Improved early graft function	Limited direct laryngeal-specific data; technical complexity
Normothermic machine perfusion	Experimental organ preservation platforms	Enables functional assessment and metabolic support during preservation	Allows *ex vivo* graft conditioning and viability assessment	Experimental stage; Not yet validated for larynx

#### 3.1.3 Immunomodulation

The larynx contains abundant antigen-presenting cells within its mucosal epithelium and submucosa, including dendritic cells and macrophages, along with dense networks of CD4⁺ and CD8⁺ T lymphocytes (36). Preclinical studies have demonstrated robust expression of major histocompatibility complex (MHC) class I and II molecules in laryngeal mucosa, rendering this region a primary target for alloimmune recognition and rejection (20, 37). Compared with cartilage and connective tissue, the mucosal compartment exhibits earlier and more pronounced immune activation following transplantation, suggesting that mucosal immunity plays a pivotal role in both acute and chronic rejection processes (9).

Importantly, ischemia–reperfusion injury further amplifies the immunogenicity of laryngeal allografts by inducing endothelial activation, upregulation of adhesion molecules, and release of damage-associated molecular patterns (DAMPs) (38, 39). These events promote leukocyte recruitment and facilitate the transition from innate to adaptive immune responses, thereby linking perioperative injury to subsequent alloimmune rejection (40).

Current immunosuppressive regimens in experimental and clinical laryngeal transplantation largely parallel those employed in other VCA procedures. Calcineurin inhibitors, particularly tacrolimus, form the backbone of most protocols and are frequently combined with corticosteroids and antiproliferative agents such as mycophenolate mofetil (41). Preclinical large-animal studies have demonstrated that tacrolimus-based regimens significantly improve short-term graft survival and reduce the severity of acute rejection episodes (37).

However, long-term systemic immunosuppression remains a major limitation of laryngeal transplantation, given that the procedure is not life-saving. Chronic exposure to immunosuppressive agents is associated with increased risks of infection, metabolic complications, nephrotoxicity, and malignancy, raising critical ethical concerns regarding risk–benefit balance (42). These considerations have driven substantial interest in immunomodulatory strategies aimed at minimizing systemic immunosuppression while preserving graft tolerance.

To mitigate the adverse effects of systemic immunosuppression, increasing attention has been directed toward targeted and local immunomodulatory approaches. Experimental studies in VCA models have explored local delivery of immunosuppressive agents directly to the graft, including topical tacrolimus application and drug-eluting biomaterials, with encouraging reductions in localized immune infiltration and epithelial injury (43, 44).

Additionally, strategies targeting specific immune pathways—such as costimulatory blockade, regulatory T cell (Treg) induction, and modulation of macrophage polarization—have demonstrated the capacity to attenuate alloimmune responses in composite tissue allografts (45–47). Although direct evidence in laryngeal transplantation remains limited, these approaches hold promise for selectively suppressing pathogenic immune activation while preserving systemic immune competence.

Effective immunomodulation in laryngeal transplantation requires not only optimized therapeutic strategies but also reliable methods for immune monitoring. The larynx offers a unique advantage in this regard, as direct endoscopic visualization and mucosal biopsy enable real-time assessment of graft status. Emerging biomarkers of immune activation, including cytokine profiles and gene-expression signatures, may further enhance early detection of rejection and guide individualized immunosuppressive adjustment (48).

In summary, immunomodulation in laryngeal transplantation must balance adequate suppression of alloimmune responses against the long-term risks of systemic immunosuppression. Future progress will likely depend on integrating perioperative injury mitigation, targeted immunomodulatory therapies, and precise immune monitoring into a comprehensive, graft-specific immunological management framework. Such advances are essential for transitioning laryngeal transplantation from experimental feasibility toward sustainable clinical application.

#### 3.1.4 Biomarkers and precision monitoring

The identification and validation of reliable biomarkers represent a critical step toward early detection and precise monitoring of immune rejection in laryngeal transplantation. As a form of vascularized composite allotransplantation (VCA), laryngeal transplantation is characterized by heterogeneous tissue composition and often exhibits subclinical immune activation before overt histopathological changes become apparent (49, 50). Consequently, traditional surveillance strategies relying primarily on clinical symptoms or histological biopsy are limited by delayed detection and procedural invasiveness, underscoring the need for sensitive and minimally invasive biomarkers capable of predicting rejection at an early stage (51).

Preclinical studies using rat laryngeal transplantation models have identified parathyroid hormone (PTH) as a potential biomarker associated with acute rejection (21). Notably, experimental evidence indicates that circulating PTH levels decline approximately 72 h prior to the onset of histologically confirmed rejection, suggesting that PTH may serve as an early predictive indicator of impending alloimmune injury. The temporal advantage conferred by this biomarker highlights its potential utility in guiding timely immunosuppressive intervention before irreversible graft damage occurs.

From a mechanistic perspective, alterations in PTH levels are unlikely to reflect direct immunological injury to the laryngeal allograft. Instead, they may represent a downstream manifestation of inflammation-mediated disruption of the endocrine–immune axis. During acute rejection, systemic inflammatory cytokine release, microvascular dysfunction, and metabolic stress may collectively interfere with parathyroid hormone synthesis, secretion, or clearance (52, 53). This observation emphasizes that endocrine biomarkers should be interpreted within a broader physiological context and supports the concept that rejection-associated biomarkers may reflect integrated systemic responses rather than isolated local tissue injury.

Given these considerations, future immune monitoring strategies in laryngeal transplantation should evolve from reliance on single biomarkers toward multidimensional, integrative assessment frameworks. Advances in multi-omics technologies provide an opportunity to expand the biomarker repertoire beyond isolated hormonal indicators (49). Inflammatory cytokine profiles—including interleukin-6 (IL-6) and tumor necrosis factor-α (TNF-α)—offer dynamic insight into immune activation intensity (54, 55), while immune cell phenotyping can inform shifts in effector and regulatory immune balance. In parallel, donor-derived cell-free DNA (dd-cfDNA) has emerged as a highly specific marker of graft injury and cellular turnover in solid organ transplantation (56, 57) and may hold similar promise for monitoring tissue damage and rejection in laryngeal allografts.

Importantly, laryngeal transplantation offers unique advantages for biomarker validation and integration. The accessibility of the laryngeal mucosa allows repeated endoscopic visualization and targeted tissue sampling, facilitating correlation of circulating biomarkers with local molecular, histological, and functional changes. Future research should prioritize cross-species validation strategies, translating candidate biomarkers identified in small-animal models into large-animal studies and ultimately clinical cohorts, thereby enhancing their robustness and translational relevance.

In summary, the development of biomarker-driven precision monitoring represents a cornerstone for the long-term success of laryngeal transplantation. By integrating multi-omics approaches, dynamic biomarker panels, and cross-species validation, postoperative management may transition from empiric immunosuppression toward data-driven, individualized immune modulation. Such progress will be essential for improving graft longevity, preserving function, and enabling the sustainable clinical application of laryngeal transplantation.

#### 3.1.5 Future directions in preclinical research

The future trajectory of laryngeal transplantation research is inherently dependent on deep multidisciplinary integration. Given the extraordinary anatomical, functional, and immunological complexity of the larynx as a vascularized composite allotransplantation (VCA) graft, progress toward reliable clinical translation can no longer rely on isolated experimental paradigms (58). Instead, coordinated advances across animal modeling strategies, immunological mechanism elucidation, data-driven analytics, bioengineering innovation, and ethical governance will be essential.

From an experimental modeling perspective, porcine models will continue to occupy a central role in surgical optimization and immunological investigation owing to their close anatomical and physiological resemblance to the human larynx. These models remain indispensable for refining microsurgical techniques, vascular reconstruction strategies, and perioperative management protocols. In parallel, rodent models offer unique advantages in genetic manipulability, particularly through CRISPR/Cas9-mediated genome editing (58, 59). Targeted modification of immune-related pathways—such as T cell receptor signaling, costimulatory molecules, or immune checkpoint regulators—provides a powerful platform for dissecting mechanisms of alloimmune recognition and immune privilege. Future research strategies that integrate the anatomical relevance of large mammals with the scalability and mechanistic precision of rodent models through cross-species or hybrid experimental designs may substantially accelerate both technical and immunological breakthroughs.

Technological convergence represents another critical frontier. The integration of high-dimensional radiomics, spatial transcriptomics, and advanced computational approaches—including machine learning and artificial intelligence algorithms—offers unprecedented opportunities to characterize spatial and temporal heterogeneity within the graft immune microenvironment (60). By correlating multimodal imaging features with spatially resolved molecular signatures, predictive models of graft viability, functional recovery, and rejection dynamics may be developed. Such data-driven frameworks have the potential to transform hypothesis generation in preclinical research and ultimately inform clinical decision-making in laryngeal transplantation.

At the translational level, the development of individualized immunosuppressive strategies is likely to be a key determinant of long-term success. Multimodal assessment incorporating biomarker panels, quantitative pharmacokinetic–pharmacodynamic modeling, and donor–recipient major histocompatibility complex (MHC) compatibility analysis may enable optimization of immunosuppressive efficacy while minimizing treatment-related toxicity (61). This precision-based approach aligns with broader trends in transplant medicine toward personalized immune modulation and may be particularly critical for laryngeal transplantation, given its non–life-saving indication and the ethical imperative to minimize long-term risk.

Advances in bioengineering further expand the landscape of preclinical innovation. Despite their promise, most bioengineering and personalized immunomodulatory strategies in laryngeal transplantation remain at the preclinical stage and currently lack robust clinical validation. Decellularized laryngeal scaffolds have emerged as a potential approach for reducing graft immunogenicity while preserving structural integrity. Recent porcine studies demonstrated that decellularization protocols can preserve extracellular matrix architecture and partially restore epithelial integrity after recellularization and implantation. Similarly, biomarker-guided immunomodulation and individualized tacrolimus adjustment remain largely investigational in laryngeal transplantation, despite encouraging findings in broader vascularized composite allotransplantation research. Therefore, these emerging approaches should currently be regarded as promising translational concepts rather than established clinical therapies. Decellularized extracellular matrix scaffolds, functionalized biomaterials, and normothermic or hypothermic machine perfusion technologies hold promise for enhancing graft tolerance, improving microvascular perfusion, and extending preservation time (56). These strategies may reduce immunogenicity and ischemic injury while providing a platform for *ex vivo* graft conditioning, thereby improving both short- and long-term outcomes. Collectively, such bioengineering approaches may also serve as stepping stones toward the development of bioartificial or hybrid laryngeal constructs with reduced immunological burden.

Concomitant with technological progress, ethical frameworks must evolve to address the increasing complexity of preclinical research. Prolonged postoperative monitoring and functional assessment in large-animal models raise important considerations related to animal welfare, resource allocation, and translational justification. Ethical oversight should therefore advance in parallel with scientific innovation to ensure responsible and sustainable research practices.

Ultimately, the future of laryngeal transplantation research will rest on the synergistic integration of surgical precision, immune regulation, and bioengineering innovation. Through coordinated multidisciplinary efforts, laryngeal transplantation may progressively transition from an experimental reconstructive approach to a standardized, predictable, and ethically justifiable clinical intervention capable of restoring function and, in selected contexts, contributing to life-saving care.

## 4 Clinical progress in laryngeal transplantation

Since the successful completion of the first human laryngeal transplantation in 1998, the field has evolved along a deliberately cautious trajectory. (Table 3) As a non–life-saving form of vascularized composite allotransplantation (VCA), laryngeal transplantation has been constrained by extraordinary surgical complexity, the risks associated with lifelong immunosuppression, and persistent ethical debate. To date, approximately 16 cases worldwide have been reported with varying degrees of documentation and academic acceptance. Although early clinical cases primarily involved traumatic or benign laryngotracheal dysfunction, oncologic laryngeal transplantation has more recently been explored in highly selected patients. However, the current evidence remains limited to a small number of heterogeneous cases, with substantial variation in indications, previous treatments, surgical techniques, functional assessment, follow-up duration, and survival reporting. Therefore, the available cases should be interpreted as evidence of technical feasibility rather than as confirmation of a definitive change in clinical practice.

**Table 3:** Reported human laryngeal transplantation cases and outcomes.

Year/country	Detailed indication	Previous treatment(s)	Reinnervation/vocal-fold findings	Voice outcome	Swallowing outcome	Airway/decannulation	LEMG/objective assessment	Follow-up, survival, and major events
1998/USA	Severe post-traumatic laryngopharyngeal stenosis and aphonia after a motorcycle accident; conventional reconstruction had failed.	Multiple previous airway and laryngeal reconstructive procedures; exact sequence NR.	SLN and RLN coaptation. Reinnervation was demonstrated, but coordinated vocal-fold abduction remained inadequate.	Early phonation was achieved; serviceable and sustained speech developed during follow-up. Objective acoustic testing was reported, although not all numerical data were consistently available.	Oral intake and functional swallowing recovered; aspiration was not a major long-term problem.	No definitive decannulation. Tracheostomy was retained because adequate glottic abduction and airway protection could not be assured.	Serial endoscopy and electrophysiological assessment/LEMG were performed; complete quantitative data NR.	Long-term graft function for approximately 14 years; subsequent graft explantation because of chronic rejection, recurrent infection, rigidity, and airway compromise. Cause of patient death NR.
2001–2006/Colombia	Traumatic or benign end-stage laryngeal dysfunction; patient-level diagnoses and severity were incompletely reported.	Previous airway or reconstructive procedures variably reported; detailed patient-level treatment histories NR.	Neural reconstruction and vocal-fold position/movement were not consistently described.	Voice outcomes were variably reported and generally lacked standardized acoustic or patient-reported measures.	Swallowing outcomes were incompletely documented; standardized instrumental or patient-reported measures NR.	Decannulation status and reasons for persistent tracheostomy were not consistently reported for individual patients.	LEMG and standardized objective assessments NR for most cases.	Survival and causes of mortality were incompletely documented at patient level; interpretation is limited by incomplete reporting.
2010/USA	Severe long-segment acquired laryngotracheal stenosis after prolonged intubation, with major airway and voice impairment.	Multiple endoscopic and open reconstructive procedures; previous kidney–pancreas transplantation and established long-term immunosuppression.	RLN and SLN reconstruction. Useful phonation returned, but functional vocal-fold abduction remained inadequate.	Speech recovered; acoustic parameters were reported as near normal during follow-up.	Pharyngeal/anastomotic stenosis required repeated dilatation and prolonged swallowing rehabilitation before oral intake was restored.	No definitive decannulation; tracheostomy was maintained because of inadequate airway opening despite useful voice.	Endoscopy and objective acoustic assessment were reported; detailed LEMG findings NR.	Long-term graft survival was reported. The patient remained alive during the available follow-up; major complications included stenosis, infection risk, and immunosuppression-related toxicity.
2015/Poland	Laryngeal squamous cell carcinoma after total laryngectomy and radiotherapy; reported as T3N1 disease in the clinical literature.	Total laryngectomy, postoperative radiotherapy, and treatment of severe postoperative wound/fistula complications; pre-existing renal transplantation and immunosuppression.	Selective motor reinnervation, including phrenic nerve-to-posterior cricoarytenoid reconstruction. Functional airway opening and phonation were restored.	Early phonation followed by useful or near-normal speech. Standardized acoustic and patient-reported voice scores were incompletely reported.	Functional oral swallowing recovered; validated instrumental and patient-reported swallowing scores NR.	Successful decannulation was reported after restoration of adequate airway patency.	Electrophysiological monitoring was reported; detailed numerical LEMG results NR.	Alive with a functioning graft during the published follow-up; no reported tumor recurrence during the reported observation period.
2023/China	Recurrent laryngeal carcinoma after previous organ-preserving surgery; detailed subsite and complete TNM stage should be reported from the primary case publication.	Previous partial laryngectomy; additional oncologic treatment details variably reported.	Selective neural preservation and/or reconstruction. Vocal-fold movement was recovering but incompletely characterized in early follow-up.	Speech and phonation improved; standardized subjective and objective voice measures were not consistently reported.	Swallowing improved and partial oral intake was restored; validated VFSS/FEES and patient-reported outcome data were limited.	Tracheostomy remained in place during early follow-up; complete decannulation had not been achieved.	Endoscopic assessment was reported; LEMG and standardized acoustic/swallowing data NR or limited.	Alive with a functioning graft in the early published follow-up; postoperative fistula/airway complications were treated. Longer-term oncologic and survival outcomes require continued follow-up.
2023/France	Irreversible long-standing laryngeal dysfunction with severe loss of voice and airway function; detailed etiology and prior procedures incompletely reported in early reports.	Multiple previous treatments were reported or implied, but the detailed sequence was NR in the initial publication.	Selective neural reinnervation/neurotization. Detailed vocal-fold position and movement were not fully reported.	Voice recovery was reported; standardized subjective and objective voice measures NR.	Swallowing recovery was incomplete in early follow-up; instrumental and patient-reported data NR.	Not decannulated during early follow-up; tracheostomy remained in place.	LEMG and standardized objective assessment NR in the initial report.	Alive with a functioning graft during early follow-up; longer-term survival and complication data were not yet available.

FEES, fiberoptic endoscopic evaluation of swallowing; LEMG, laryngeal electromyography; NR, not reported; RLN, recurrent laryngeal nerve; SLN, superior laryngeal nerve; VFSS, videofluoroscopic swallowing study.

The landmark case performed at the Cleveland Clinic in 1998 is widely regarded as the foundation of clinical laryngeal transplantation. The recipient was a 40-year-old man with severe post-traumatic laryngopharyngeal stenosis following a motorcycle accident, for whom conventional reconstructive options had failed. The allograft included the entire larynx along with the thyroid gland, parathyroid glands, and several tracheal rings. Postoperatively, the patient achieved meaningful functional recovery, including normal swallowing and sustained phonation for more than 12 years. Episodes of acute rejection were transient and successfully managed with cyclosporine-based immunosuppression (9, 62). This case provided the first compelling evidence that laryngeal transplantation could restore complex laryngeal functions in humans, while simultaneously highlighting the substantial technical and logistical barriers to widespread clinical adoption.

Subsequently, the University of Antioquia in Colombia reported the largest single-center experience to date, claiming 13 laryngeal transplantations performed between 2001 and 2006, with a reported 2-year graft survival rate of 90% and only two cases of acute rejection (63). The authors further suggested that recipients could tolerate perioperative hemodynamic instability without strict fluid management. However, critical details—including vascular anastomotic techniques, nerve reconstruction strategies, long-term functional outcomes (such as voice quality), and immunosuppressive protocols—were insufficiently reported. The absence of transparent, verifiable data and the lack of independent replication led to widespread skepticism within the international community. Consequently, these 13 cases were largely excluded from global consensus between 2007 and 2024. Only in October 2024 did Estephania Candelo, in a multinational review spanning three continents, provisionally recognize five of these cases based on verifiable evidence (64). This episode underscored the urgent need for standardized reporting and rigorous evidence validation in the field.

In 2010, the University of California, Davis expanded the potential indications for laryngeal transplantation through a unique clinical scenario. The recipient was a 51-year-old woman with severe laryngotracheal stenosis secondary to prolonged intubation, who had previously undergone combined kidney–pancreas transplantation and was already receiving long-term immunosuppressive therapy. This pre-existing immunological context partially mitigated ethical concerns regarding lifelong immunosuppression. Although a tracheostomy was maintained postoperatively, the patient regained oral breathing and phonation, demonstrating the feasibility of laryngeal transplantation in patients already committed to immunosuppressive treatment (65). This case suggested that baseline immunological status may become an important factor in future patient selection.

A major conceptual shift occurred in 2015, when the Gliwice Cancer Center in Poland performed laryngeal transplantation in a 34-year-old patient with laryngeal squamous cell carcinoma who had previously undergone total laryngectomy and radiotherapy. Remarkably, spontaneous breathing and phonation were restored within days after transplantation (66). This case marked the first clinical application of laryngeal transplantation in an oncologic setting. However, it also raised profound concerns regarding the potential for immunosuppression to promote tumor recurrence, thereby emphasizing the necessity for stringent patient selection and long-term oncologic surveillance. The case exemplified the dual nature of laryngeal transplantation in cancer rehabilitation, in which functional restoration and oncologic risk coexist.

Since 2023, oncologic laryngeal transplantation has been investigated at West China Hospital in a carefully selected group of patients with advanced or recurrent laryngeal and hypopharyngeal malignancies. Although institutional reports referred to seven procedures, complete peer-reviewed patient-level data were not available for all seven recipients. A subsequently published pilot series described four patients who underwent laryngeal transplantation between 2023 and 2024. (67) Recipient selection incorporated oncologic resectability, the absence of distant metastasis, medical suitability for lifelong immunosuppression, rehabilitation potential, and psychosocial assessment, although the detailed scoring criteria and consent framework were not fully reported. Patient age, sex, tumor subsite, TNM stage, previous treatment, operative characteristics, functional recovery, decannulation status, disease-free survival, graft survival, and patient survival are summarized in Table 4 where available.

**Table 4:** Patient-level characteristics and outcomes of four oncologic laryngeal transplantations performed at west China hospital between 2023 and 2024.

Case	Age/sex	Tumor subsite and stage	Previous treatment	Transplant/donor/operative details	Functional outcomes	Decannulation	Follow-up, DFS, graft status, and survival
Case 1	65/male	Recurrent laryngeal SCC, rT3N0M0	Right vertical partial laryngectomy 9 years previously; emergency tracheostomy. Additional chemoradiotherapy was not reported.	Composite laryngeal allotransplantation with bilateral vascular reconstruction and SLN/RLN anastomosis. Individual donor age/sex and patient-level operative time were not reported.	Swallowing recovery was delayed by postoperative pharyngoesophageal dysfunction/diverticulum and required secondary endoscopic treatment. Semiliquid oral intake was subsequently achieved. Standardized acoustic outcomes and LEMG were not reported.	No. The tracheostoma remained in place during follow-up.	Follow-up/DFS/graft survival: 10 months. The patient developed severe pulmonary and systemic infection with sepsis and died 10 months after transplantation.
Case 2	52/male	Hypopharyngeal SCC, T3N3M0	Emergency tracheostomy; two cycles of albumin-bound paclitaxel, cisplatin, and tislelizumab as neoadjuvant treatment.	Composite transplantation using the refined extracorporeal-perfusion protocol with bilateral vascular and laryngeal nerve reconstruction. Individual donor details and operative time were not reported.	Soft-diet intake was achieved during follow-up. Detailed vocal-fold motion, validated voice assessment, instrumental swallowing scores, and LEMG were not reported.	No. The patient remained tracheostoma-dependent.	Tumor recurrence occurred at 7 months. The patient died at 11 months after transplantation; death was associated with recurrent disease (fatal tracheostomy-site hemorrhage was reported).
Case 3	53/male	Laryngeal SCC, T4aN0M0	Emergency tracheostomy; two cycles of albumin-bound paclitaxel plus cisplatin as neoadjuvant treatment.	Narrow-field tumor resection followed by composite transplantation; extracorporeal HTK perfusion; bilateral superior thyroid vascular anastomoses and bilateral SLN/RLN anastomoses. Individual donor details were not reported.	Progressive neural recovery occurred at approximately 4–5 months. Vocal folds changed from a paramedian to a more medial position. Complete oral intake was achieved by 6 months. Voice improved markedly; reported MPT was 18.49 s and S/Z ratio 1.22.	No. Bilateral medial vocal-fold positioning supported phonation but produced respiratory distress during tracheostomy occlusion.	Alive at 20-month follow-up; DFS 20 months. Rejection-free survival was initially 20 months. Graft rejection developed after self-discontinuation of immunosuppressive therapy and required renewed treatment.
Case 4	54/male	Recurrent laryngeal SCC, rT4aN0M0	Previous rigid-laryngoscopic laser surgery and emergency tracheostomy; two cycles of albumin-bound paclitaxel, cisplatin, and cadonilimab as neoadjuvant treatment.	Composite transplantation with extracorporeal HTK perfusion and bilateral vascular, SLN, and RLN reconstruction. Individual donor details and operative time were not reported.	Progressive RLN recovery was observed at approximately 4–5 months. Complete oral intake was achieved by 6 months and maintained. Laryngoscopy demonstrated vestibular-fold movement and vocal-fold vibration. Reported MPT was 11.69 s and S/Z ratio 1.62.	No. Respiratory distress occurred during tracheostomy occlusion because of bilateral medial vocal-fold positioning.	Alive with a functioning graft at 26-month follow-up; DFS 26 months and rejection-free survival 23 months. No recurrence or metastasis was reported at the latest follow-up.

DFS, disease-free survival; HTK, histidine–tryptophan–ketoglutarate; LEMG, laryngeal electromyography; MPT, maximum phonation time; RLN, recurrent laryngeal nerve; SCC, squamous cell carcinoma; SLN, superior laryngeal nerve.

Outcomes in this pilot cohort were heterogeneous. Follow-up ranged from 10 to 26 months. Two recipients remained alive with functioning grafts and achieved meaningful recovery of phonation and swallowing; however, both remained dependent on a tracheostomy, indicating that recovery of voice and oral intake did not equate to restoration of an independent upper airway. One surviving recipient developed acute graft rejection after independently discontinuing immunosuppressive therapy. The remaining two patients died at 10 and 11 months after transplantation, respectively, one from severe pneumonia complicated by sepsis and the other from tumor recurrence. These findings demonstrate technical feasibility but also illustrate substantial oncologic, infectious, immunological, and airway-related risks. Accordingly, the current evidence should be interpreted as an early single-center pilot experience rather than proof that oncologic laryngeal transplantation provides durable oncologic control or predictable functional independence.

The oncologic experience remains difficult to generalize because of the small cohort size, concentration of cases within a single institution, short and unequal follow-up, and incomplete reporting of several clinically relevant variables. Detailed information regarding donor–recipient anatomical matching, donor sex, psychosocial eligibility criteria, consent procedures, LEMG findings, validated acoustic and swallowing assessments, operative duration, and procedural cost was not consistently available. Furthermore, persistent tracheostomy dependence in the surviving recipients indicates that satisfactory phonation and swallowing should not be interpreted as complete restoration of laryngeal function. Prospective multicenter reporting using predefined oncologic eligibility criteria and standardized functional and survival outcomes is required before the procedure can be considered reproducible or generalizable.

In September 2023, the French ECLAT team performed the first laryngeal transplantation in France for a patient with irreversible laryngeal dysfunction due to trauma, further extending the geographic reach of this technique (68, 69). Although functional outcomes such as voice quality remain under evaluation, this case reinforced the role of laryngeal transplantation in trauma rehabilitation and demonstrated its feasibility within European transplant frameworks.

Despite these advances, significant challenges remain. The contrast between the limited transparency of the Colombian series and the detailed, reproducible reports from centers in the United States, Poland, France, and China highlights the urgent need for standardized outcome metrics, including validated voice and swallowing assessment tools, as well as long-term immunosuppression monitoring networks. In oncologic applications, future protocols must balance tumor control—taking into account tumor stage and biological behavior—with precisely tailored immunosuppressive strategies. The preliminary success of individualized immunosuppression based on tumor biology reported by the Chinese team offers a critical reference paradigm for this emerging field.

### 4.1 Donor selection in laryngeal transplantation

The success of laryngeal transplantation critically depends on meticulous donor selection, with the overarching goal of balancing anatomical compatibility, immunological safety, and preservation of graft integrity. As a form of vascularized composite allotransplantation (VCA), laryngeal transplantation involves the survival of multiple tissue components—including mucosa, cartilage, muscle, nerves, and vasculature—and directly determines the restoration of highly refined functions such as phonation, swallowing, and respiration. Consequently, the stringency of donor selection criteria plays a decisive role in long-term graft survival and functional outcomes.

Early clinical experience established foundational principles for donor selection. In the first successful total laryngeal transplantation performed in 1998, the donor was a 40-year-old male who died from rupture of a cerebral aneurysm. The donor had no history of smoking, was seronegative for cytomegalovirus (CMV) and Epstein–Barr virus (EBV), and was fully matched with the recipient at human leukocyte antigen (HLA) loci (9). This highly favorable donor–recipient pairing was considered instrumental in reducing acute rejection risk and ensuring durable graft function, and it strongly influenced subsequent perceptions of an “ideal” donor profile in laryngeal transplantation.

Building on this experience, Duque and colleagues proposed a more standardized donor selection protocol, specifying donor age between 18 and 50 years, ABO blood group compatibility, absence of smoking or substance abuse history, intubation duration of less than 3 days, and intensive care unit (ICU) stay shorter than 7 days (63). Given the larynx's marked sensitivity to ischemia, mechanical injury, and infection, these parameters were designed to minimize structural damage and immunological activation, thereby improving transplantation success.

Despite the value of stringent donor criteria, real-world clinical practice often requires a pragmatic balance between ideal conditions and donor availability. This flexibility is exemplified by the 2010 laryngeal transplantation case reported by the University of California, Davis. In this case, the donor was a 38-year-old male who was CMV-seropositive and exhibited partial HLA mismatches (including 2 A, 1 B, 1 Cw, 1 DR, and 1 DQ alleles) (65). Notably, the recipient had previously undergone combined kidney–pancreas transplantation and had demonstrated good tolerance to long-term immunosuppression, allowing successful compensation for suboptimal donor matching. This case suggests that a recipient's pre-existing immunosuppressive status may serve as a modulating factor in future donor selection strategies, potentially permitting greater flexibility in HLA matching.

Similarly, the Colombian research group cautiously utilized donors with a history of smoking or marijuana use in situations where alternative donors were unavailable. In their reported series of 13 laryngeal transplantations, donors were predominantly young males with a mean age of 27.2 ± 7.9 years, and the most common cause of death was severe head trauma (63). Although some donors had a history of mild substance use, the selection process prioritized donors with fewer systemic comorbidities, reflecting a pragmatic emphasis on overall health status and anatomical integrity rather than absolute exclusion based on single risk factors.

In the Chinese oncologic cohort, donor identification and screening were conducted in accordance with the institutional protocols of the Red Cross Society of China and the Ethics Committee of West China Hospital. Eligible donors were brain-dead individuals selected on the basis of ABO blood-group compatibility and anthropometric matching to ensure appropriate laryngeal size, airway caliber, and graft fit. Donors with a history of head and neck malignancy, previous laryngeal disease or trauma, structural abnormalities of the larynx, or active systemic infection were excluded. Pre-procurement assessment included evaluation of laryngeal anatomy and mucosal integrity, together with immunological compatibility testing and a negative crossmatch.

Regardless of evolving criteria, anatomical and functional compatibility remains the cornerstone of donor selection in laryngeal transplantation. Pre-procurement laryngoscopic evaluation is considered essential for excluding laryngotracheal injury, inflammation, or structural abnormalities. In the Colombian series, donors with normal laryngeal anatomy and intact mucosa were preferentially selected to maximize the likelihood of functional recovery after transplantation (63).

Procurement techniques have progressively evolved toward *en bloc* retrieval of the pharyngolaryngeal complex, including the larynx, thyroid gland, parathyroid glands, selected tracheal rings, and key vascular structures such as the superior thyroid artery and internal jugular vein, while preserving associated neural elements whenever possible (62). Nevertheless, technical challenges remain significant. For example, in the 1998 landmark case, intraoperative injury to the left internal jugular vein necessitated adaptive vascular anastomotic strategies to maintain adequate graft perfusion. Such experiences underscore the critical importance of advanced microsurgical expertise and intraoperative decision-making during donor procurement.

Looking ahead, the future of donor selection in laryngeal transplantation lies in optimizing the dynamic balance between rigorous standards and clinical flexibility. In selected recipient populations—particularly those already receiving long-term immunosuppression—moderate relaxation of age limits or tolerance of minor HLA mismatches may help expand the donor pool without substantially increasing immunological risk. In parallel, advances in *ex vivo* organ perfusion technologies, especially normothermic machine perfusion, may extend ischemic tolerance beyond the current threshold of approximately 20 h, thereby alleviating logistical constraints associated with donor procurement and transportation (70).

Innovations in bioengineering, including decellularized extracellular matrix scaffolds and partial laryngeal transplantation techniques, may further reduce reliance on whole cadaveric donors, particularly in patients with a high risk of tumor recurrence (71). However, as procurement strategies expand and technical complexity increases, ethical frameworks must evolve accordingly. Concerns regarding donor body integrity, procurement timing, and the extent of surgical retrieval often complicate informed consent processes for donor families, necessitating transparent communication and robust ethical oversight.

### 4.2 Surgical techniques in laryngeal transplantation

Laryngeal transplantation represents the pinnacle of reconstructive surgery, demanding exceptional precision in microvascular and neural anastomosis, donor–recipient anatomical matching, and multidisciplinary perioperative collaboration. A complete procedure typically includes en-bloc procurement of the donor pharyngolaryngeal complex with back-table preparation, resection of the scarred or diseased larynx in the recipient, vascular and neural reconstruction, and subsequent airway and digestive tract reconstruction with functional assessment (72, 73). The first human laryngeal transplantation performed at the Cleveland Clinic in 1998 established fundamental operative principles. Through a combined cervical and suprasternal approach, the pharyngolaryngeal complex—including the larynx, thyroid gland, parathyroid glands, and several tracheal rings—was harvested *en bloc*, with preservation of key vascular structures such as the superior thyroid artery and internal jugular vein to facilitate later anastomosis (72). Early clinical attempts relied primarily on end-to-end anastomosis of the superior thyroid arteries and internal jugular veins to secure arterial inflow and venous outflow (73). With increasing experience, the University of California, Davis group refined vascular reconstruction by performing end-to-side anastomosis of the donor innominate vein to the recipient internal jugular vein and prioritizing revascularization of the superior thyroid artery, thereby reducing cold ischemia time to approximately 300 min and improving mucosal perfusion and edema control (65). Other centers reported technical flexibility by selecting alternative inflow and outflow vessels according to anatomical conditions—for example, using the transverse cervical or subclavian arteries when superior thyroid artery exposure was difficult and favoring the internal jugular vein in most venous reconstructions (64, 74). Nevertheless, intraoperative vascular injury, such as damage to the left internal jugular vein in the first human transplant, remains a significant technical challenge requiring adaptive reconstruction strategies (72).

The central technical challenge of laryngeal transplantation is not merely graft survival but the restoration of coordinated functions of breathing, phonation, and swallowing through neural reinnervation. In the first human case, microanastomosis of the donor superior laryngeal and recurrent laryngeal nerves to their recipient counterparts led to the progressive return of voluntary phonation and protective swallowing reflexes, with electrophysiological evidence of functional reinnervation (74). Early strategies relying on simple direct coaptation often resulted in variable outcomes due to scarring, poor-quality nerve ends, and misdirected axonal regrowth. Evidence from animal models and bilateral vocal fold paralysis management indicates that non-selective reinnervation may fail to restore adequate abduction and may paradoxically induce inspiratory adduction (75). This understanding promoted selective neural reinnervation strategies. Notably, the 2015 Polish case used phrenic nerve grafts directed to the posterior cricoarytenoid muscle, providing respiration-coupled activation that improved glottic abduction and airway patency. Additional approaches employing the ansa cervicalis, hypoglossal branches, or selective adductor branch reinnervation further illustrate a shift from simple reconnection of nerves to intentional reconstruction of functional neural circuits centered on airway protection and phonatory control (76, 77).

Tracheal and upper airway reconstruction is another technically demanding step with direct implications for airway stability and timing of decannulation. In the 1998 case, the “parachute technique” was used to reconstruct the posterior tracheal wall first, followed by anterior and lateral wall closure, to achieve precise alignment under limited exposure. Later experience emphasized tension-free end-to-end anastomosis and overall stabilization of the laryngotracheal complex, often combined with hyoid or thyroid cartilage suspension to minimize anastomotic traction, graft descent, and stenosis, while facilitating earlier restoration of oral breathing (12). Depending on patient-specific anatomy, some centers incorporated cartilage reinforcement or interposition grafts to prevent anastomotic collapse, while maintaining airway patency and endoscopic accessibility.

Like other vascularized composite allografts, the larynx is highly vulnerable to ischemia–reperfusion injury. Early cases reported total ischemia times exceeding 18 h, and isolated European cases approached 27 h, which correlated with edema, airway compromise, and chronic rejection risk (78, 79). Explantation of the first laryngeal transplant after 14 years due to chronic rejection and graft failure has been partially attributed to prolonged cold preservation and reperfusion injury leading to fibrosis and structural remodeling (62, 80). Consequently, recent strategies have focused on shortening cold ischemia through synchronized donor–recipient procedures, optimizing static cold storage solutions, and exploring hypothermic or normothermic machine perfusion. Studies across vascularized composite allotransplantation domains demonstrate improved endothelial integrity, better microcirculatory flow, reduced apoptosis, and extended preservation windows with machine perfusion compared with static storage (81, 82).

Overall, surgical techniques in laryngeal transplantation are evolving from feasibility demonstration toward precision-driven standardization. (Table 5) Emphasis has shifted toward minimizing ischemia time, improving quality of neural reinnervation, optimizing airway reconstruction, and preventing chronic rejection. With continuing integration of microsurgical techniques, neural regeneration biology, and ex-vivo preservation technologies, laryngeal transplantation is expected to achieve further advances in safety, reproducibility, and long-term functional outcomes.

**Table 5:** Surgical techniques in laryngeal transplantation.

Surgical component	Technical approach	Key observations	Clinical significance	Limitations/challenges
Donor graft procurement	En bloc retrieval of the pharyngolaryngeal complex including larynx, thyroid and parathyroid glands, and tracheal rings	Preserves vascular, neural, and endocrine integrity; Facilitates composite graft viability	Enables physiological reconstruction and endocrine preservation	Technically demanding; Risk of vascular injury during procurement
Vascular reconstruction	End-to-end or end-to-side anastomosis of superior thyroid artery and internal jugular or innominate vein	Adequate perfusion achievable with flexible vessel selection; Shortened ischemia improves mucosal viability	Determines early graft survival and edema control	Limited vessel length; Intraoperative vascular injury risk
Neural reinnervation (motor)	Coaptation of recurrent laryngeal nerve or selective reinnervation (e.g., phrenic nerve to PCA muscle)	Selective strategies improve airway patency and respiratory coordination	Critical for restoration of vocal fold motion and decannulation	Risk of synkinetic reinnervation; Trade-off between airway and phonation
Neural reinnervation (sensory)	Anastomosis of superior laryngeal nerve	Enhances laryngeal sensation and protective reflexes	Reduces aspiration risk and improves swallowing safety	Sensory recovery is variable and often delayed
Tracheal and airway reconstruction	Tension-free end-to-end anastomosis; Parachute technique; Laryngotracheal suspension	Stable airway alignment achievable; Facilitates earlier oral breathing	Essential for airway stability and decannulation	Anastomotic stenosis and collapse may require secondary procedures
Ischemia time management	Synchronized donor–recipient surgery; Rapid revascularization	Shorter ischemia associated with reduced edema and chronic fibrosis	Improves long-term graft durability	Logistically complex; Limited preservation window
Adjunctive stabilization techniques	Cartilage reinforcement or suspension procedures	Reduces graft descent and airway collapse	Improves structural stability	Adds operative complexity

PCA, posterior cricoarytenoid muscle.

### 4.3 Postoperative management after laryngeal transplantation

Laryngeal transplantation offers a paradigm-shifting reconstructive option for patients with irreversible laryngeal dysfunction, enabling simultaneous restoration of phonation, swallowing, and airway function. However, the long-term success of this procedure depends critically on meticulous postoperative management, particularly in the domains of immunosuppressive therapy, functional rehabilitation, infection prophylaxis, and rigorous long-term surveillance. Compared with solid organ transplantation, postoperative management after laryngeal transplantation must also account for the unique immunobiology of mucosa-rich composite tissues and the functional complexities of reinnervated muscles and articulating cartilages (83).

#### 4.3.1 Immunosuppressive regimens

Immunosuppression remains the cornerstone of graft survival following laryngeal transplantation. Since the first human laryngeal transplant in 1998, immunosuppressive strategies have evolved substantially. In that landmark case, the regimen consisted of cyclosporine, mycophenolate mofetil, methylprednisolone, and OKT3 (muromonab-CD3). However, nephrotoxicity and hypertension developed, and after an episode of acute rejection 15 months postoperatively, cyclosporine was replaced with tacrolimus to improve both efficacy and toxicity profiles (72). Subsequent experience has confirmed tacrolimus as the backbone of maintenance immunosuppression in laryngeal and other vascularized composite allotransplantation (VCA) procedures due to its superior T-cell inhibitory activity and improved rejection control compared with cyclosporine (84–86).

A major advance in immunosuppressive management in this field has been the transition from steroid-dependent to steroid-sparing or steroid-free protocols when clinically feasible. For example, in the 2010 University of California, Davis case, tacrolimus-based therapy combined with leflunomide permitted long-term graft survival in a recipient who had previously undergone kidney–pancreas transplantation and experienced BK polyomavirus activation, thereby enabling stable graft function under steroid-free conditions for more than a decade (65). Similarly, the 2015 Polish laryngeal transplantation case in a prior renal transplant recipient utilized a regimen comprising tacrolimus, rabbit antithymocyte globulin (rATG), and mycophenolate mofetil, permitting complete avoidance of corticosteroids while maintaining excellent graft function. These cases underscore the growing emphasis on individualized immunosuppression balancing immunologic control against systemic toxicity.

Induction therapy has also undergone significant refinement. Whereas early protocols relied on OKT3, which is associated with substantial cytokine release and neurologic toxicity, more recent strategies favor polyclonal antithymocyte globulin (ATG) or interleukin-2 receptor antagonists to reduce early rejection while minimizing adverse events (87). Maintenance therapy generally follows a triple-drug approach consisting of tacrolimus, mycophenolate mofetil, and low-dose corticosteroids; however, regimen modification is often required in patients with prior organ transplantation, viral reactivation risk, or drug intolerance (Table 6).

**Table 6:** Immunosuppressive strategies used in laryngeal transplantation and vascularized composite allotransplantation.

Regimen	Mechanism	Clinical role	Advantages	Major limitations
Tacrolimus	Calcineurin inhibitor	Mainstay maintenance therapy	Strong T-cell suppression; reduced acute rejection	Nephrotoxicity, neurotoxicity, infection risk
Cyclosporine	Calcineurin inhibitor	Early maintenance protocols	Historically established	Inferior rejection control compared with tacrolimus
Mycophenolate mofetil (MMF)	Antiproliferative agent	Combination maintenance therapy	Synergistic immunosuppression	Bone marrow suppression, gastrointestinal toxicity
Corticosteroids	Broad anti-inflammatory effect	Induction and maintenance	Rapid rejection control	Metabolic complications, osteoporosis
rATG	T-cell depletion	Induction therapy	Potent early immunosuppression	Cytokine release, infection risk
Leflunomide	Pyrimidine synthesis inhibitor	Alternative maintenance therapy	Useful in viral-risk recipients	Hepatotoxicity, hematologic toxicity
Local tacrolimus delivery	Local immunomodulation	Experimental adjunct strategy	Reduced systemic exposure	Limited clinical evidence
Costimulatory blockade	Targeted immune modulation	Experimental	Potential reduction in chronic rejection	Limited translational data

Despite these advances, complications remain frequent. Chronic rejection continues to pose a major barrier to durable graft survival. In vascularized composite allotransplant cohorts, chronic rejection rates as high as 20%–30% have been reported, often associated with subtherapeutic immunosuppressant levels or poor adherence (80). In laryngeal transplantation, the mucosal component of the graft—with its abundance of antigen-presenting cells—appears particularly vulnerable to chronic inflammatory remodeling and fibrosis. Opportunistic infections represent another dominant challenge. Cytomegalovirus (CMV) reactivation, Pneumocystis jirovecii pneumonia, and fungal infections have been reported after laryngeal and other composite tissue transplantation, necessitating the use of prophylactic regimens such as trimethoprim–sulfamethoxazole and ganciclovir or valganciclovir in high-risk recipients (88).

Consequently, contemporary postoperative management emphasizes therapeutic drug monitoring of tacrolimus trough levels, routine viral surveillance (CMV, BK, EBV), regular endoscopic evaluation of the laryngeal mucosa, and protocol biopsies when feasible to detect subclinical rejection. Future directions include the integration of donor-derived cell-free DNA, gene-expression profiling, and non-invasive imaging biomarkers to improve sensitivity for early rejection detection while minimizing biopsy-associated morbidity (56).

Overall, effective postoperative care following laryngeal transplantation requires dynamic integration of personalized immunosuppressive adjustment, infectious disease prophylaxis, rigorous rejection monitoring, and coordinated functional rehabilitation. These strategies are essential to convert surgical success into sustained functional recovery and long-term graft survival.

#### 4.3.2 Functional recovery after laryngeal transplantation

Functional recovery after laryngeal transplantation is highly heterogeneous and reflects the interplay between surgical technique, the success of neural reinnervation, immune stability, graft microstructure integrity, and adherence to structured rehabilitation programs. Unlike solid organ transplantation, the primary endpoint in laryngeal transplantation extends beyond graft survival to include coordinated restoration of phonation, swallowing, and airway protection.

Clinical outcomes after laryngeal transplantation should be reported across distinct functional domains rather than summarized using general terms such as “functional recovery.” These domains should include vocal-fold position and mobility, electrophysiological evidence of reinnervation, subjective and objective voice assessment, instrumental and patient-reported swallowing assessment, airway patency, decannulation status, and the need for secondary airway procedures. Importantly, restoration of useful phonation or oral intake does not necessarily indicate recovery of coordinated vocal-fold abduction or successful decannulation. Patient survival, graft survival, chronic rejection, tumor recurrence, and causes of mortality should therefore be reported separately.

Standardized outcome assessment is essential for evaluating functional recovery after laryngeal transplantation. Future reports should move beyond descriptive terms such as “voice recovery” or “improved swallowing” and adopt predefined, reproducible outcome measures. Voice outcomes should include both objective acoustic parameters, such as fundamental frequency, jitter, shimmer, harmonic-to-noise ratio, and maximum phonation time, and validated patient-reported instruments such as the Voice Handicap Index-10 (VHI-10) or Voice-Related Quality of Life (V-RQOL). The VHI-10 was developed and validated as an abbreviated voice handicap instrument, and a clinically meaningful improvement has commonly been interpreted using minimal important difference or minimal clinically important difference thresholds in voice-disorder populations.

Swallowing outcomes should be assessed using both instrumental and functional measures. Recommended tools include videofluoroscopic swallowing study or fiberoptic endoscopic evaluation of swallowing, the Penetration–Aspiration Scale (PAS), the Functional Oral Intake Scale (FOIS), the Eating Assessment Tool-10 (EAT-10), and the MD Anderson Dysphagia Inventory (MDADI). The PAS provides an 8-point scale for quantifying airway invasion during swallowing, while FOIS evaluates functional oral intake and EAT-10 provides a validated symptom-specific dysphagia assessment.

Decannulation should be reported as a distinct endpoint rather than being merged with general airway improvement. Standardized decannulation outcomes should include decannulation rate, time to decannulation, duration of tracheostomy-free survival, airway patency on endoscopy, need for secondary airway procedures, and recurrence of airway obstruction. Quality-of-life assessment should incorporate both generic and disease-specific instruments, such as the Short Form-36, EQ-5D, University of Washington Quality of Life questionnaire, and EORTC QLQ-C30 combined with the head-and-neck module. The EORTC head-and-neck module has been validated in laryngectomized patients and includes domains directly relevant to swallowing, speech, social eating, and social contact.

Current evidence indicates a general temporal sequence of recovery—swallowing function precedes phonation, which in turn precedes decannulation—although the trajectory varies substantially among recipients.

Swallowing function typically begins to recover within 3–6 months postoperatively. Early impairment is commonly attributable to anastomotic stenosis, supraglottic edema, sensory denervation, and delayed cortical motor relearning. In the first human laryngeal transplantation, complete oral intake was achieved at 14 weeks, and the use of glycopyrrolate to reduce salivary pooling facilitated rehabilitation (72). Conversely, in the 2010 University of California, Davis case, pharyngeal anastomotic stenosis necessitated repeated dilations and prolonged dysphagia therapy for 11 months before functional swallowing was restored (89). Videofluoroscopic and high-resolution manometry studies suggest that inadequate cricopharyngeal relaxation, delayed epiglottic inversion, and impaired laryngeal sensation constitute major physiological substrates for aspiration after transplantation (90).

Voice recovery is even more dependent on the pattern and quality of neural reinnervation. Acoustic analyses demonstrate that fundamental frequency, jitter, shimmer, and maximum phonation time frequently normalize within 12–24 months; however, persistent restriction of vocal fold mobility is common secondary to incomplete or misdirected laryngeal reinnervation (9, 64). In the landmark 1998 case, “serviceable speech” was achieved by 36 months, yet incomplete recurrent laryngeal nerve recovery resulted in inadequate glottic abduction and long-term tracheostomy dependence. Similarly, the 2010 case exhibited near-normal acoustic parameters at 18 months without restoration of functional vocal fold abduction, necessitating permanent tracheostomy (89). These observations underscore that restoration of intelligible speech does not equate to complete motor recovery of the larynx.

The marked variability in clinical outcomes after laryngeal transplantation is likely multifactorial. Recipient-related factors, including baseline airway anatomy, scar burden, prior tracheostomy, previous reconstruction, radiotherapy, nutritional status, neuromuscular reserve, and rehabilitation capacity, may substantially influence postoperative recovery. In oncologic recipients, prior radiotherapy and extensive ablative surgery may further impair vascular quality, wound healing, and functional restoration. Donor–recipient anatomical compatibility and graft quality are also important, as size mismatch, mucosal injury, intubation-related damage, and prolonged cold ischemia may contribute to edema, impaired mucosal viability, fibrosis, and unstable airway outcomes. Surgical technique represents another key determinant. Although vascular reconstruction is essential for graft survival, functional recovery depends largely on precise neural reinnervation and airway stabilization. Non-selective recurrent laryngeal nerve coaptation may restore phonation but fail to achieve coordinated vocal fold abduction, whereas selective phrenic nerve–posterior cricoarytenoid reinnervation may improve airway patency but provide limited phonatory control. Immunological factors, including acute rejection, chronic inflammation, infection, and suboptimal immunosuppression, may further promote fibrosis and functional decline. Finally, differences in rehabilitation protocols and outcome reporting hinder comparison across cases. Therefore, future studies should adopt standardized multidomain outcomes covering voice, swallowing, decannulation, airway function, quality of life, rejection, and secondary procedures.

Selective neural reinnervation strategies have emerged as a major innovation. Phrenic-nerve–to–posterior-cricoarytenoid reinnervation produces respiration-coupled abduction and has shown promise for airway patency, particularly in preventing inspiratory obstruction (65). Clinical application within the ECLAT consortium demonstrated successful decannulation and improved ventilatory function after phrenic nerve implantation, although phonatory quality improved only modestly, indicating potential trade-offs between robust airway opening and fine phonatory control (64). In parallel, attempts at superior laryngeal nerve sensory reinnervation have been associated with reductions in aspiration and enhancement of protective reflexes, further highlighting the importance of sensory-motor integration in functional outcomes (9).

Decannulation remains the final and most demanding functional milestone. Recent multinational cohort data indicate that only approximately 50% of recipients achieve tracheostomy closure within two years following transplantation (64). Persistent supraglottic edema, cicatricial stenosis, synkinetic reinnervation, and glottic insufficiency are leading causes of decannulation failure and often necessitate secondary interventions, including laser aryepiglottoplasty, posterior cordotomy, or arytenoid fixation procedures. These findings reinforce that laryngeal transplantation should be conceptualized as a multistage reconstructive process, wherein secondary procedures constitute integral components rather than complications alone.

In summary, functional recovery after laryngeal transplantation depends on the coordinated reconstruction of anatomy, neural circuitry, and neuromotor learning. Future directions include objective multimodal assessment frameworks combining acoustic analysis, swallowing videofluoroscopy, electromyography, and high-resolution imaging; electrophysiologically guided selective reinnervation; neuromodulatory rehabilitation platforms; and artificial intelligence–based individualized functional prognostication models. These strategies are expected to support the transition from graft survival to durable, high-quality laryngeal function as the principal therapeutic goal.

#### 4.3.3 Long-term surveillance after laryngeal transplantation

Long-term postoperative surveillance is essential for early detection of rejection, timely management of complications, and preservation of graft function after laryngeal transplantation. Because the larynx is a mucosa-rich vascularized composite allograft, it is particularly susceptible to both acute and chronic rejection, necessitating systematic, multimodal monitoring strategies (91, 92).

Direct endoscopic visualization combined with mucosal biopsy remains the current gold standard for rejection assessment. Endoscopic features of acute rejection typically include diffuse mucosal edema, erythema, ulceration, epithelial sloughing, or abnormal secretions (12). Histopathology commonly demonstrates lymphocytic epithelial infiltration, basal layer degeneration, endothelialitis, and submucosal edema, paralleling rejection patterns described in facial and limb vascularized composite allotransplantation (92). However, repeated biopsy carries the risk of bleeding, supraglottic scarring, and airway compromise, prompting development of minimally invasive alternatives.

Metabolic and endocrine monitoring has been incorporated into some laryngeal transplant programs. The Polish team introduced systematic assessment of thyroid-stimulating hormone (TSH), parathyroid hormone (PTH), and serum calcium to evaluate thyroid–parathyroid complex function when these glands are transplanted together with the larynx. Nevertheless, this approach is less informative in recipients retaining native glands. Nuclear medicine techniques, including radioiodine scintigraphy to evaluate thyroidal iodine uptake, provide functional imaging of transplanted endocrine tissue and may aid differentiation between hypofunction and rejection-related inflammation.

Recent advances in transplant immunology have promoted the integration of noninvasive immunologic biomarkers into surveillance protocols. Detection of donor-specific anti-HLA antibodies (DSA) has emerged as an important predictor of antibody-mediated rejection and chronic graft vasculopathy in both solid organ and composite tissue allotransplantation (93). In parallel, donor-derived cell-free DNA, peripheral cytokine signatures, and transcriptomic profiling of mucosal swabs are being investigated as early indicators of immune activation that may precede histologic changes. These methods hold promise for reducing biopsy frequency while enabling earlier therapeutic intervention.

Chronic rejection represents the most serious long-term threat to graft survival. Characteristic features include progressive submucosal fibrosis, cartilage ankylosis, and obliterative microangiopathy, ultimately resulting in graft stiffening and irreversible functional decline. Registry data from vascularized composite allotransplantation cohorts suggest that up to 20%–30% of patients may experience chronic rejection, often several years after initially successful transplantation (94). This pattern was exemplified by the first human laryngeal transplant recipient, who required graft explantation after 14 years due to progressive rigidity, recurrent infection, and airway compromise, despite prolonged initial functional success (62). These findings emphasize the necessity of lifelong, structured surveillance rather than limited short-term follow-up.

Oncologic surveillance constitutes another essential component of long-term management. Prolonged systemic immunosuppression is associated with increased risk of virus-related and *de novo* malignancies, including post-transplant lymphoproliferative disease and human papillomavirus (HPV)–related squamous cell carcinoma of the aerodigestive tract (95, 96). Because the transplanted larynx contains mucosal tissue directly exposed to environmental carcinogens, and because HPV has a recognized role in laryngeal dysplasia and carcinoma, vigilance for mucosal neoplasia is imperative. Regular endoscopic inspection, HPV testing when indicated, and rapid biopsy of suspicious lesions are recommended.

Beyond rejection and malignancy, airway complications, endocrine dysfunction, chronic infections, and immunosuppression toxicity also mandate continued follow-up. Multidisciplinary surveillance combining otolaryngology, transplant immunology, endocrinology, speech-language pathology, and infectious disease expertise is now regarded as standard of care (94).

Taken together, long-term management after laryngeal transplantation must evolve toward integrated precision monitoring frameworks incorporating endoscopy, functional testing, immunologic biomarkers, advanced imaging, and longitudinal patient-reported outcomes. These strategies are essential for preventing late irreversible graft failure and for securing durable quality-of-life benefits in this non–life-saving yet profoundly life-altering procedure.

From a critical perspective, the current clinical evidence should be regarded as preliminary rather than definitive. The total number of reported human laryngeal transplantations remains extremely small, and the quality of reporting varies considerably among centers. Some reports provide detailed descriptions of vascular reconstruction, neural repair, immunosuppression, rejection episodes, and functional outcomes, whereas others lack sufficient methodological transparency. This inconsistency makes direct comparison difficult and prevents reliable estimation of graft survival, rejection incidence, decannulation rate, voice quality, and swallowing recovery. Moreover, successful cases are more likely to be published, which may introduce publication bias and overestimate the true clinical effectiveness of the procedure. Therefore, while existing cases support feasibility, they do not yet establish reproducibility, safety, or long-term superiority over conventional rehabilitation strategies.

## 5 Ethical considerations and evaluation in laryngeal transplantation

To contextualize the unique challenges of laryngeal transplantation within the broader field of vascularized composite allotransplantation, Table 7 compares laryngeal transplantation with face and hand transplantation in terms of functional objectives, immunological characteristics, rehabilitation requirements, and ethical considerations. Laryngeal transplantation, as a form of vascularized composite allotransplantation (VCA), raises a set of ethical questions that differ fundamentally from those associated with life-saving solid organ transplantation. Whereas heart, liver, or kidney transplantation is primarily intended to prolong survival, the principal objective of laryngeal transplantation is restoration of phonation, swallowing, and airway-related functions, thereby improving social participation and health-related quality of life (97, 98). Consequently, ethical evaluation must carefully balance long-term risks of systemic immunosuppression against anticipated functional and psychosocial benefits within a non–life-saving therapeutic context.

**Table 7:** Comparison of laryngeal transplantation with face and hand vascularized composite allotransplantation.

DomainE1:H10	Laryngeal transplantation	Face transplantation	Hand transplantation
Primary indication	Irreversible laryngeal dysfunction	Severe facial disfigurement	Bilateral upper-limb loss
Main functional goal	Respiration, phonation, swallowing	Facial expression, appearance, speech	Motor and sensory restoration
Life-saving	No	No	No
Identity-bearing organ	High (voice and communication)	Very high (facial identity)	Moderate
Tissue composition	Mucosa, cartilage, muscle, nerves	Skin, muscle, bone, nerves	Bone, tendon, muscle, nerves
Immunologic risk	High mucosal antigenicity	High skin antigenicity	High skin antigenicity
Chronic rejection risk	Significant	Significant	Significant
Major long-term concern	Airway stability and chronic rejection	Psychosocial adaptation	Motor recovery durability
Ethical challenge	Immunosuppression for non–life-saving function restoration	Identity and resource allocation	Risk–benefit proportionality

### 5.1 Risk–benefit balance in a non–life-saving transplant

Recipients of laryngeal transplants must commit to lifelong immunosuppression with attendant risks, including opportunistic infection, metabolic complications, nephrotoxicity, and increased incidence of malignancy (99). In life-saving transplantation, these risks are often ethically justified as proportional to the benefit of preserved survival. In contrast, the ethical calculus is more complex for laryngeal transplantation, where the primary expected benefit is improvement in voice, swallowing ability, airway function, and social reintegration. Many total-laryngectomy patients experience profound communication restriction, social isolation, body-image disturbance, and depression; their quality-of-life impairment has been reported to rival that seen in advanced cancer (92). Thus, the key ethical question is not whether the procedure prolongs life, but whether the degree of functional recovery and restoration of identity is sufficient to justify permanent systemic risk. Current ethical frameworks recommend limiting laryngeal transplantation to carefully selected patients with severe persistent disability after conventional treatments who demonstrate full understanding of potential risks and uncertain benefit (93).

A key unresolved ethical issue is whether the expected improvement in voice, swallowing, airway function, and social participation can justify lifelong systemic immunosuppression in a non–life-saving procedure. Unlike solid organ transplantation, laryngeal transplantation requires a more individualized assessment of proportionality, because functional recovery may be delayed, incomplete, or limited to specific domains. Therefore, ethical justification should depend not only on surgical feasibility, but also on the likelihood of meaningful patient-centered benefit. Informed consent should be regarded as an ongoing process that includes discussion of uncertain outcomes, alternative rehabilitation options, possible secondary procedures, long-term surveillance, and the psychological implications of receiving a donor larynx. In addition, the identity-bearing nature of voice requires sensitive counseling for both recipients and donor families. Given the resource-intensive nature of this procedure, laryngeal transplantation should be performed only in specialized centers with independent ethics review, strict patient selection, transparent reporting, and long-term multidisciplinary follow-up.

### 5.2 Special ethical issues in oncologic indications

The extension of laryngeal transplantation from traumatic and benign indications to selected laryngeal and hypopharyngeal cancer survivors introduces additional ethical complexity. Candidates must simultaneously meet criteria for oncologic safety—such as locoregional disease control, absence of distant metastasis, and favorable tumor biology—and tolerability of immunosuppression without unacceptable risk of recurrence (93).

For oncologic indications, patient selection should be particularly stringent because lifelong immunosuppression may increase the risk of tumor recurrence or secondary malignancy. Candidates should have localized or locoregionally controlled disease, no distant metastasis, acceptable tumor biology, severe functional impairment, and limited conventional reconstructive options. Risk stratification should consider tumor stage, nodal status, HPV status, histological aggressiveness, margin status, recurrence pattern, prior radiotherapy, feasibility of complete tumor clearance, immunosuppression tolerance, infection risk, nutritional status, adherence, psychosocial readiness, and willingness to undergo lifelong surveillance. Thus, oncologic laryngeal transplantation should remain a highly selective procedure rather than a routine reconstructive option.

Although transplantation may dramatically improve communication, swallowing, and social functioning in cancer survivors, immunosuppression is a well-recognized promoter of virus-related and *de novo* malignancies, including HPV-associated squamous cell carcinoma and post-transplant lymphoproliferative disorders. Ethical evaluation therefore requires explicit consideration of tumor recurrence risk based on stage, HPV status, histopathology, molecular subtype, and longitudinal imaging surveillance. Recent clinical experiences employing strict oncologic selection criteria, individualized immunosuppression minimization, and intensive post-transplant monitoring have achieved encouraging early outcomes, but limited sample sizes preclude broad generalization. The prevailing ethical stance remains that laryngeal transplantation for oncologic indications should be regarded as a highly selective reconstructive option, reserved for carefully evaluated patients with compelling functional need and informed consent.

### 5.3 Informed consent and psychosocial assessment

Informed consent for laryngeal transplantation is inherently complex and goes beyond standard surgical disclosure. Patients must understand not only procedural risks but also: 1. The inevitability of lifelong immunosuppression and its systemic complications; 2. The possibility of incomplete or delayed functional recovery, including persistent tracheostomy dependence; 3. The likelihood of secondary interventions for stenosis, edema, or chronic rejection; 4. Potential disturbances in personal identity related to using another person's larynx and producing voice through a donor organ (94).

Contemporary VCA guidelines emphasize comprehensive psychological and psychosocial evaluation of all candidates to assess treatment adherence, coping capacity, family support, and risk of substance misuse or psychiatric instability (100–102). Equally important is assessment of the individual's readiness to accept changes in voice quality and neck appearance, as these are closely tied to self-image and social identity.

### 5.4 Donor issues, resource allocation, and justice

Because laryngeal transplantation relies on deceased donors, it necessarily involves ethical questions surrounding donor authorization and organ allocation. Prevailing consensus holds that laryngeal retrieval should occur only after allocation of life-saving organs has been secured and within transparent organ procurement frameworks with explicit authorization for visible organ retrieval. The larynx, like the face or hands, is a highly identity-bearing organ; donor families may experience heightened psychological distress related to perceived loss of voice or personal identity of the deceased. Sensitive counseling and targeted consent processes are therefore required, with clear discussion of bodily integrity, dignity, and the specific nature of laryngeal donation (103).

Moreover, laryngeal transplantation is resource-intensive, technically complex, and currently benefits a relatively small patient population. Questions of distributive justice arise concerning prioritization of healthcare resources and reimbursement. Several authors argue that laryngeal transplantation should be conceptualized similarly to hand and face transplantation—as selective, reconstructive, and research-stage therapy—and should proceed only where it does not compromise access to essential medical care and occurs under robust ethical and regulatory oversight.

In light of these considerations, many centers advocate structured ethical decision-making pathways for laryngeal transplantation within broader VCA governance frameworks. Recommended elements include: 1. Confirmation of irreversible functional impairment and failure of conventional reconstruction; 2. Individualized oncologic risk assessment where applicable; 3. Comprehensive psychological and social evaluation; 4. Independent institutional ethics committee review of each case; Long-term prospective follow-up and transparent reporting through registries or multicenter databases.

The ethical justification of laryngeal transplantation remains highly dependent on the strength of clinical evidence. At present, the balance between functional benefit and systemic risk is difficult to quantify because standardized patient-reported outcomes, quality-of-life measurements, and long-term comparative data are lacking. This issue is particularly important in oncologic recipients, for whom immunosuppression may theoretically increase the risk of tumor recurrence or secondary malignancy. Although early reports are encouraging, the follow-up duration and sample size are insufficient to confirm oncologic safety. Therefore, future studies should move beyond descriptive case reporting and adopt prospective registries, standardized outcome metrics, transparent reporting of complications, and long-term surveillance protocols.

### 5.5 Comparison with standard treatment and rehabilitation

Ethical evaluation of laryngeal transplantation should be framed against the current standard of care, particularly total laryngectomy with tracheoesophageal voice-prosthesis rehabilitation. Total laryngectomy offers reliable oncologic control, airway stability, and aspiration prevention, while voice prostheses can restore functional communication. However, patients remain dependent on a permanent stoma and require lifelong prosthesis maintenance, replacement, and speech rehabilitation, with risks of leakage, biofilm formation, and device-related complications.

Laryngeal transplantation aims to restore respiration, phonation, swallowing, sensation, and upper-airway continuity more physiologically. Nevertheless, current evidence is insufficient to show consistent superiority over modern post-laryngectomy rehabilitation. Some recipients recover speech and oral intake but remain tracheostomy-dependent because of impaired vocal-fold abduction, stenosis, edema, or synkinesis.

Unlike total laryngectomy, laryngeal transplantation requires lifelong immunosuppression and carries risks of infection, nephrotoxicity, metabolic complications, malignancy, rejection, and graft loss. Because it is primarily a quality-of-life procedure, candidate selection must carefully consider expected functional benefit, comorbidities, treatment adherence, psychological resilience, rehabilitation capacity, and willingness to accept lifelong surveillance.

### 5.6 Cost, resource use, and equality of access

Direct cost-effectiveness comparisons between laryngeal transplantation and standard post-laryngectomy rehabilitation are not yet available. Total laryngectomy with voice-prosthesis rehabilitation involves ongoing costs for prosthesis replacement, stoma care, heat-and-moisture exchange devices, speech therapy, and management of device-related complications. Although these costs may limit access for some patients, the treatment is well established within existing head-and-neck oncology services.

Laryngeal transplantation is likely to require substantially greater resources because it involves donor procurement, complex microsurgery, intensive care, lifelong immunosuppression, repeated monitoring, and prolonged multidisciplinary rehabilitation. Its availability is also restricted to a few specialized centers, creating potential geographic, socioeconomic, and insurance-related inequalities. Transparent selection criteria, equitable referral pathways, ethical oversight, and formal economic evaluation are therefore essential.

Psychosocial outcomes also require careful assessment. Transplantation may improve voice, communication, self-image, and social participation, but it may also cause anxiety related to rejection, cancer recurrence, graft failure, and lifelong treatment. Total laryngectomy can likewise lead to stigma, communication difficulties, and social isolation. These effects should be evaluated longitudinally using validated psychosocial measures.

### 5.7 Coordination of donor availability with oncologic treatment

Oncologic laryngeal transplantation requires careful coordination between donor availability and cancer treatment. Because oncologic resection should not be delayed while awaiting a suitable graft, candidates should undergo complete staging, assessment of resectability and surgical margins, evaluation of metastatic risk, prior treatment review, immunological and psychosocial assessment, and donor–recipient anatomical matching within a multidisciplinary framework.

After approval, patients may be conditionally listed and rapidly reassessed when a compatible donor becomes available. Resection and transplantation can then be performed in a coordinated procedure, provided that adequate oncologic margins remain achievable. Donor availability should never influence the extent of tumor removal or delay treatment beyond an acceptable interval. If no suitable graft is available in time, standard treatment, including total laryngectomy and conventional rehabilitation, should proceed.

Because donor grafts and specialized resources are limited, allocation criteria should be transparent and consider oncologic prognosis, expected functional benefit, adherence, anatomical compatibility, and resource use. Until stronger long-term evidence is available, oncologic laryngeal transplantation should remain limited to prospective protocols with independent ethical oversight and registry-based follow-up.

## 6 Limitations

This review has several limitations. First, the clinical evidence remains limited because only a small number of human laryngeal transplantation cases have been reported worldwide, most of which are isolated case reports or small single-center experiences. Second, substantial heterogeneity exists in patient indications, donor selection, surgical techniques, reinnervation strategies, immunosuppressive regimens, rehabilitation protocols, follow-up duration, and outcome assessment. These differences limit direct comparison across studies and prevent reliable estimation of graft survival, rejection incidence, decannulation rate, voice recovery, swallowing function, and quality-of-life improvement. Third, reporting quality varies considerably, with some cases lacking detailed or independently verifiable data on surgical procedures, immunosuppression, complications, and long-term functional outcomes. In addition, several emerging strategies discussed in this review, including machine perfusion, biomarker-guided monitoring, local immunomodulation, and bioengineered scaffolds, remain largely preclinical or extrapolated from broader vascularized composite allotransplantation research. Finally, long-term oncologic safety under lifelong immunosuppression remains uncertain. Future studies should establish multicenter registries, standardized outcome measures, and transparent long-term reporting of both successful and unsuccessful cases.

## 7 Conclusion

In summary, although laryngeal transplantation has progressed from an experimental concept to a clinically feasible therapeutic strategy, its broader application still depends on deep multidisciplinary integration. Future advances will require the convergence of transplant immunology, bioengineering innovation, microsurgical technique, and rehabilitation medicine, in order to overcome key barriers such as improving long-term graft survival, reducing the burden of lifelong immunosuppression, and optimizing comprehensive health management for recipients. Continued refinement in immune modulation, personalized perioperative care, neural reinnervation, and functional rehabilitation protocols will be essential to translate laryngeal transplantation from highly selected clinical use toward a safer, more durable, and widely accessible reconstructive option for patients with irreversible laryngeal dysfunction.
